# Epidemiology, clinical features, therapeutic interventions and outcomes of mucormycosis in Shiraz: an 8-year retrospective case study with comparison between children and adults

**DOI:** 10.1038/s41598-022-21611-8

**Published:** 2022-10-13

**Authors:** Marjan Motamedi, Zahra Golmohammadi, Somayeh Yazdanpanah, S. Mojtaba Saneian, Mojtaba Shafiekhani

**Affiliations:** 1 Department of Medical Parasitology and Mycology, School of Medicine, Shiraz University of Medical Sciences, Shiraz, Iran; 2 Department of Clinical Pharmacy, Faculty of Pharmacy, Shiraz University of Medical Sciences, Shiraz, Iran; 3 Shiraz Organ Transplant Center, Abu-Ali Sina Hospital, Shiraz University of Medical Sciences, Shiraz, Iran; 4 Shiraz Transplant Research Center, Shiraz University of Medical Sciences, Shiraz, Iran

**Keywords:** Diseases, Risk factors, Signs and symptoms

## Abstract

Mucormycosis is an invasive fungal infection with high morbidity and mortality rate despite the early diagnosis and proper therapeutic interventions. Given the importance of epidemiological data in reviewing the attitude toward infectious diseases in developing countries, the current retrospective case study aimed to compare the epidemiological aspects, risk factors, clinical characteristics, therapeutic interventions, and outcomes of mucormycosis between adults and children during eight years (2013–2021) in the main infectious disease referral centers in the southwest of Iran. The median age of 164 patients included in this study was 47 years (IQR 22–59). The median length of hospitalization was 33 days.The annual incidence of mucormycosis-related hospitalizations was estimated 1.76 per 10,000 admissions during the study period. Moreover, the incidence of infection was 2.4 times higher in males than females in children. Diabetes mellitus was the most frequent predisposing factor in adults (46.0%). The main risk factor in children was hematologic malignancy (52.6%), but a considerable proportion of them (28.9%) were immunocompetent.The most frequent antifungal agent used was liposomal amphotericin B (82.3%) as monotherapy. The combination therapy was used more in adults (15.8%) than children (7.9%). In addition, surgical intervention with antifungal therapy was considered the most effective therapeutic approach. The in-hospital mortality rate was 14.6% for adults, whereas it was zero for children. Our findings provide a recent epidemiologic analysis of mucormycosis among hospitalized patients in both children and adults. Mucormycosis mainly affects individuals with diabetes mellitus or hematological malignancies and presents as rhino-orbito-cerebral form. Proven diagnosis of mucormycosis according to clinical manifestations and histopathology observations accompanied by proper antifungal treatments may improve survival rates.

## Introduction

Fungal infections by Mucoralean fungi, namely mucormycosis, are serious and progressive diseases that get increasingly worse^1^. Mucormycosis affects all age groups, and is associated with significant morbidity and death in both immunocompromised and immunocompetent hosts, depending on the underlying risk factors, site of infection, and early management^2^.

Studies from all over the world indicate uncontrolled diabetes and hematologic malignancies are predominant risk factors for mucormycosis. The other common predisposing conditions associated with mucormycosis include solid organ/hematopoietic stem cell transplantation, neutropenia, a prolonged period of steroid therapy, treatment with iron chelators such asdeferoxamine, extensive burns, and traumatic events^3^.

Despite the extended dispersion of Mucorales spores in the environment, most species are not capable of infecting humans because they could not proliferate at body temperature. Humans can acquire the infection predominantly through inhalation of spores, occasionally by ingestion of contaminated food or traumatic inoculation. After germination of the spores, sprout branch-like hyphae invade blood vessels and cause necrosis of the surrounding tissues^4,5^.

The most infected sites in mucormycosis are paranasal sinuses; however, lungs, skin, and brain are other organs that can be infected with mucormycosis. Either the Gastrointestinal system or kidneys are other internal organs that are less likely to become involved^6^.

Standard approaches for the treatment of mucormycosis include a combination of antifungal therapy and surgical intervention to remove infected tissues, especially in rhino-orbito-cerebral form, and control the underlying condition. Liposomal amphotericin B (LAMB) is the first choice that is preferable to the conventional form due to less toxicity and side effects. Moreover, such triazoles as posaconazole or isavuconazole have shown desirable antifungal activity against mucormycosis^7^.

The epidemiology of mucormycosis has been investigated in previous studies in different world regions, so it has been reported that the prevalence of mucormycosis is about 0.16 cases per 10,000 discharges among the American population^8^. Although, the prevalence of mucormycosis is estimated 50 times higher than global data in developing countries, such as Mexico and India^9,10^. Recently, there has been an increase in the incidence of the infection which is linked to increased immunocompromised patients and the presence of various underlying conditions^3^. Although the number ofcase reports inchildren is growing^11^, there is not acomprehensive study comparing the epidemiological pattern of mucormycosisin adults and children population. In this regard, the aim of this retrospective case study is the comparison of epidemiological aspects, risk factors, clinical characteristics, diagnosis, therapeutic interventions, and outcomes of mucormycosis between adults and children for eight years (2013–2021) in the main infectious disease referral hospitals in the southwest of Iran as the one of an endemic zone in the Middle East.

## Results

### Patient’s characteristics

In total, 164 patients with confirmed mucormycosis were included in this study among 927,404 total hospitalized patients during the study period. The number of patients in children and adults population was 38 (23.2%) and 126 (76.8%) cases, respectively.

Of patients, 91 (55.5%) were male and 73 (44.5%) were female (male/female ratio: 1.2:1). Median age of all patients included in this study was 47 years (IQR 22–59). The mean age of adult patients (> 18 years old) was 51.83 ± 15.21, and the mean age of patients in children group (< 18 years old) was 8.10 ± 5.40 years. The median length of stay in hospital was 33 days.

In children, the incidence of infection was 2.4 times higher in males than in females, while this ratio was almost the same in the adult population. The adult to children mucormycosis ratio was 3.3:1.

According to our results, the annual incidence (annual infection rate) of mucormycosis-related hospitalizations was estimated to be 1.76 per 10,000 admissions during the study period. Moreover, the annual incidence of mucormycosis varied from 2.78 per 10,000 admissionsin 2016 to 1.04 per 10,000 admissions in 2020. During the study period, an increase in the incidence of mucormycosis was observed in autumn (n:52, 31.7%). Demographics and characteristics of patients in the total population and also, based on the age group classification (adults/children) are provided in Table 1.

**Table 1:** Demographic and clinical characteristics of adultand children patients with confirmed mucormycosis (n = 164).

	Total (N = 164)	Children (N = 38)	Adults (N = 126)	p-value
**Gender**				
Male	91 (55.5%)	27 (71.1%)	64 (50.8%)	0.04
Female	73 (44.5%)	11 (28.9%)	62 (49.2%)	
**Seasonal distribution**				
Spring	26 (15.9%)	9 (23.7%)	17 (13.5%)	0.14
Summer	48 (29.3%)	10 (26.3%)	38 (30.2%)	
Autumn	52 (31.7%)	11 (28.9%)	41 (32.5%)	
Winter	38 (23.2%)	8 (21.1%)	30 (23.8%)	
**Cases per year**				
2013–2014	20 (12.2%)	1 (2.6%)	19 (15.1%)	0.53
2014–2015	15 (9.1%)	4 (10.5%)	11 (8.7%)	
2015–2016	13 (7.9%)	1 (2.6%)	12 (9.5%)	
2016–2017	22 (13.4%)	7 (18.4%)	15 (11.9%)	
2017–2018	19 (11.6%)	5 (13.2%)	14 (11.1%)	
2018–2019	33 (20.1%)	12 (31.6%)	21 (16.7%)	
2019–2020	27 (16.5%)	6 (15.8%)	21 (16.7%)	
2020–2021	15 (9.1%)	2 (5.3%)	13 (10.3%)	
**Site of infection**				
Rhino-orbito-cerebral	158 (96.4%)	37 (97.4%)	121 (96%)	0.61
Pulmonary	2 (1.2%)	1 (2.6%)	1 (0.8%)	
Gastrointestinal	1 (0.6%)	–	1 (0.8%)	
Rare forms^†^	3 (1.8%)	–	3 (2.4%)	
**Predisposing risk factors**				
Hematological malignancies	62 (37.8%)	18 (47.4%)	44 (34.9%)	< 0.001
AML	35	5	30	
ALL	17	9	8	
CLL	6	0	6	
Non-Hodgkin lymphoma	4	4	0	
Diabetes mellitus	61 (37.2%)	3 (7.9%)	58 (46.0 %)	
Iatrogenic trauma	5 (3.0%)	1 (2.6%)	4 (3.2%)	
Chronic Corticosteroid use	5 (3.0%)	–	5 (4.0%)	
Solid organ transplantation	4 (2.4%)	–	4 (3.2%)	
Chemotherapy	1 (0.6%)	–	1 (0.8%)	
OtherPredisposing factor^‡^	7 (4.3%)	3 (7.9%)	4 (3.2%)	
Immunocompetent patients	18 (11.0%)	11 (28.9%)	7 (5.6%)	
**Clinical manifestation**				
Pain and swelling of the eye	45 (27.4%)	15 (39.5%)	30 (23.8%)	0.60
Bloody nasal discharge with obstruction in the nasal cavities	30 (18.3%)	6 (15.8%)	24 (19.0%)	
Headache	28 (17.1%)	7 (18.4%)	21 (16.7%)	
Swelling and facial pain	26 (15.9%)	3 (7.9%)	23 (18.3%)	
Decreased level of consciousness	14 (8.5%)	2 (5.3%)	12 (9.5%)	
Fever	9 (5.5%)	2 (5.3%)	7 (5.6%)	
Nasal cavity and septum ulcer	5 (3.0%)	1 (2.6%)	4 (3.2%)	
Hard palate ulcer	4 (2.4%)	1 (2.6%)	3 (2.4%)	
Facial/ abducens nerve palsy	3 (1.8%)	–	3 (2.4%)	
Cough and shortness of breath	2 (1.2%)	1 (2.6%)	1 (0.8%)	
Abdominal pain	1 (0.6%)	–	1 (0.8%)	
**Treatment**				
LAMB^§^	141 (86.0%)	35 (92.1%)	106 (84.2%)	0.05
LAMB and posaconazole	7 (4.2%)	3 (7.9%)	4 (3.1%)	
LAMB with other triazoles	10 (6.1%)	–	10 (8.0%)	
LAMB with azole and caspofungin	6 (3.7%)	–	6 (4.7%)	
**Outcome**				
Survival	136 (82.9%)	38 (100%)	98 (77.8%)	< 0.001
Mortality	24 (14.6%)	–	24 (19.0%)	
Discharge with personal consent	4 (2.4%)	–	4 (3.2%)	

^†^Rare forms include iatrogenic mucormycosis, abscess formation in the neck and pterygoid muscle.

^‡^Other Predisposing factors include chronic kidney disease, thalassemia major and long-term use of Deferoxamine.

^§^*LAMB* Liposomal amphotericin B.

### Site of infection

During this study, the most common site of mucormycosis was rhino-orbito-cerebral involvement which manifest in 158 (96.4%) patients. Excep than one gastrointestinal mucormycosis reported in one adult patient, rhino-orbito-cerebral involvement was the only clinical form that was obsereved in other patients with no underlying conditions (n:17, 94.4%).

Pulmonary mucormycosis were reported in 2 patients (1.2%) with diabetes mellitus: one child with tracheal involvement, and another case in an adult with obstruction of the right main bronchus and small lesions of the carina in the left main bronchus. In one case of gastrointestinal mucormycosis in an adult patient, infection was confined to the terminal ileum, cecum, appendix, and right colon. Although rare clinical forms of mucormycosis were not observed in children, they were reported in 3 adult patients in this study as follows: one case of iatrogenic mucormycosis resulted in fungus ball formation in the brain following surgery,  one case of abscess formation in the neck and lower jaw, and one caseof abscess formation in the pterygoid muscle. Also,the most common form of infection in children population of this study was rhino-orbito-cerebral mucormycosis (n:37, 97.4%).

### Predisposing risk factors

In the total population of our study, the main predisposing factor for mucormycosis were hematological malignancies (n:62, 37.8%) and diabetes mellitus (n:61, 37.2%).

Statistical analysis of data showed a significant relationship between predisposing factors and the age grouping of patients (p-value < 0.001). Diabetes mellitus (n:58, 46.0%) and hematological malignancies (n:44, 34.9%) were the most frequent predisposing factors in adults. Hematological malignancies (n:20, 52.6%) were the main risk factor in children, althougha high proportion of children (n:11, 28.9%) in our study were immunocompetent or no risk factor was found among them.

Of those patients with hematological malignancy, AML was diagnosed in 35 cases (21.3%) following with ALL (n:17, 10.4%), CLL (n:6, 3.6%), with non-Hodgkin Lymphoma (n:4, 2.4%).

Chronic corticosteroid use (n:5, 3%), iatrogenic trauma (n:5, 3%) following tooth extraction or maxillofacialsurgery, and solid organ transplantation (n:4, 2.4%) were other risk factors. Moreover, chronic kidney disease (CKD), thalassemia major, and long-term use of Deferoxamine were reported as underlying conditions in 7 (4.3%) patients.

### Findings of clinical manifestation

Pain, swelling, and protrusion of the eye (n:45, 27.4%) were the most common symptoms among the rhino-orbito-cerebral forms of mucormycosis at the time of diagnosis, followed by dirty or bloody nasal discharge (n:30, 18.3%), with or without obstruction in the nasal cavities. Significant reduction in the vision of the affected eye was considered a complication of mucormycosis in 3 patients with rhino-orbito-cerebral form. Other symptoms of rhino-orbito-cerebral mucormycosis included headache (n: 28, 17.1%), swelling and facial pain (n:26, 15.9%), decreased level of consciousness (n:14, 8.5%), fever (n:9, 5.5%), nasal cavity and nasal septum ulcer (n:5, 3%), and hard palate ulcer (n:4, 2.4%), respectively. Among those who presented facial pain and swelling, two patients had facial nerve palsy, and one had abducens nerve palsy. Cough, shortness of breath, and hemoptysis were common symptoms of pulmonary mucormycosis. In the only case of gastrointestinal mucormycosis reported in this study, abdominal pain was the most important symptom of the infection.

### Findings of radiological diagnosis

Radiological evidence was used as an initial diagnostic evaluation in patients who suspected mucormycosis. According to radiological data, thickening of sinusoidal mucosa was observed in 96.3% of the rhino-orbito-cerebral forms of mucormycosis cases. Moreover, complete obstruction of sinuses, bone erosion or destruction, presence of retro-orbital mass, and invasion of nerves or ocular tissue was reported as common clinical features of mucormycosis are associated with sinusoidal and rhino-orbito-cerebral involvements. After the thickening of sinusoidal mucosa, the presence of retro-orbital mass was one of the radiological observations that were significant in the children population (n:10, 26.3%). Radiological assessment of other forms of mucormycosis cases declared the presence of mass or abscess in the infected tissue. Also, inflammation of the mucosal membrane or the presence of nodules was reported in flexible bronchoscopy in gastrointestinal involvement.

### Findings of laboratory diagnosis

Laboratory-based diagnosis of infection including histopathological examination on biopsy of the necrotic mass or mucosal membrane was performed with H&E and/or PAS staining. In this regard, interstitial inflammation with non-septate fungal hyphae was remarked in reported results.

### Treatment

The therapeutic approaches for the treatment of our cases included sinus and ocular decompression, mass resection of the affected sites, control of underlying factors including diabetes and neutropenia, and intravenous antifungal therapy. Table 2 shows various antifungal therapeutic regimens used in our patients. All patients underwent surgical intervention except for 2 cases because of old age and critical condition of the patient's disease.

**Table 2:** Outcomes according to antifungal regimen in adults and children with confirmed mucormycosis (n = 164).

	LAMB^†^		LAMB and posaconazole		LAMB with other triazoles		LAMB with azole and caspofungin	
	Survival	Mortality	Survival	Mortality	Survival	Mortality	Survival	Mortality
Children	35 (92.1%)	–	3 (7.9%)	–	–	–	–	–
Adults	87 (69.0%)	16 (12.6%)	2 (1.6%)	2 (1.9%)	5 (4.0%)	4 (3.1%)	4 (3.1%)	2 (1.6%)
p value	< 0.001		0.28		–		–	

^†^*LAMB* Liposomal amphotericin B.

The most frequently antifungal agent used was Liposomal Amphotericin B (LAMB) as monotherapy in 141 (86%) patients of the total population. Table 2 shows different therapeutic regimens among children and adults. Combination therapy with LAMB and triazoles were prescribed in 17cases (10.3%) followed by: LAMB + posaconazole (n:7), LAMB + fluconazole (n:6), LAMB + voriconazole(n:2), and LAMB + itraconazole (n:2). Antifungal combination treatment with LAMB, posaconazole, and caspofungin was applied for 2 cases (1.2%). Also, caspofungin was used as a combination therapy in 4 cases treated with LAMB and triazoles. In children, the mean prescribed dose for LAMB was 8.9 ± 1.10 mg/kg/day. Overall, there is a statistically significant relationship between the two populations and the type of drug received for treatment (P-value = 0.05), such that the combination therapy was used more in adults (n:20, 15.8%) than children (n:3, 7.9%). Based on statistical analysis, the mortality of adult patients who received LAMB alone (16/106, 15%) for management the mucormycosis was significantly less than those treated with combination therapy (8/20, 40%) (p-value = 0.013).

### Clinical outcomes

Survival to hospital discharge was considered the primary outcome. According to the results, the rate of survival to hospital discharge in our study was 82.9%. The in-hospital mortality rate in the total population was 14.6%. Moreover, the mortality rate in adults was 19%, whereas it was zero in children (p-value < 0.001).

Regarding clinical signs, adult patients with a decreased level of consciousness showed a poor prognosis and a significant correlation with mortality (p-value < 0.001).

Patients with rhino-orbito-cerebral mucormycosis had the highest mortality rate (n:14, 19.6%), while no mortality was reported among other clinical forms of mucormycosis such as pulmonary, gastrointestinal, and rare clinical forms of mucormycosis.

## Discussion

Mucormycosis is an invasive fungal infection in immunocompromised patients with high mortality despite early diagnosis and timely therapeutic intervention. During the two last decades, the studies have shown an increase in mucormycosis compared to other invasive mycoses such as aspergillosis among people withimmune deficiencies^12^. To the best of our knowledge, this research is one of the largest studies that provides a comparison of epidemiology and clinical aspects of mucormycosis between children and adults in a developing country such as Iran.

Although mucormycosis has a worldwide occurrence, its frequency is variable in different world regions.The exact incidence of mucormycosis is not clear, and this was partly because there are a few population-based studiesthat differ in capture periods, populations, and definition or diagnostic procedures^13^. According to the previous study by Kontoyiannis et al., which was conducted in the United States, the incidence of mucormycosis-related hospitalizations was estimated to be 0.12 per 10,000 discharges from January 2005 to June 2014^14^. However, a recent study has declared an 80 times higher prevalence of mucormycosis in India than those developed countries^15^. From the results of this study, the annual incidence of mucormycosis was 1.76 per 10,000 hospitalized cases, which is a little more than that reported by Hedayati et al. from Iran in 2018^16^.Our results support the data previously reported by Vaezi et al. that has presented Fars province (Shiraz) as the second province in Iran in terms of the high prevalence of mucormycosis^17^.

Regarding demographic characteristics of our patients who suffered mucormycosis, we showed the occurrence of the disease in a wide spectrum of ages from childhood to old age. According to previous reports, mucormycosis is a rare infection in children; hence, there are few case studies in the literature^11,18^. In the current study, children consist of approximately one-fourth (23.2%) of the total populationwith mucormycosis which was more than in the previous study^11^.

In agreement with the investigation by Vaezi et al. average age of cases in this study was almost 40 years^17^, while the reported mean age varies from 50 to 60 years in other studies in Iran^19–21^. A male predominance was noted during the study similar to most of the other previous studies^6,17,20,21^; however, the sex difference was significant among children. Some authors have correlated this tendency with the protective effect of estrogens, but this correlation may not be valid in children younger than 12 years old^11^.

The prevalence of mucormycosis in our study is higher in autumn in accordance with other investigations^19,22,23^. As declared previously, environmental factors such as temperature and humidity are affected the mold distribution in the air resulting in seasonal variation of exposure to spores^23,24^.

Based on studies in different parts of Iran and other countries in the world, diabetes mellitus enumerates as the most common underlying conditions for mucormycosis^6,17,19,23,25–28^. In accordance with previous investigations, the current study showed diabetes mellitus is the most relevant predisposing factor for mucormycosis in adults.

Furthermore, investigations admitted differences in the epidemiology of mucormycosis between developed and developing countries which declared patients with hematological malignancies are at greater risk for mucormycosis in Europe and the United States^29–32^. Considering our results, hematologic malignancies and diabetes mellitus are identified almost in equal amounts as the most important underlying conditions in the development of mucormycosis. Since neutrophils have played a crucial role in the innate immune function that is responsible for engulfing and killing pathogenic microbes and invading the germinated form of *Mucorales* spores, reduced chemotactic activity in diabetic patients and prolonged neutropenia in hematologic malignancies favor the formation of hyphal structures and resulted in the development of mucormycosis in these vulnerable groups^33^. Overall, any disorders or organ failure could emerge as a potential factor for mucormycosis. In this regard, we found that impairment in the human immune system in patients receiving corticosteroid and solid organ transplant recipients or iatrogenic inoculation could be considered a predisposing factor for mucormycosis similar to other investigations^3,6,17^.

Based on our findings, rhino-orbito-cerebral form was the most common form of mucormycosis in both population that are in accordance with previous literatures^3,10,17^. One major finding in our study is that a significant proportion of mucormycosis has been reported in immunocompetent hosts (n:18, 11.0%) in agreement with other previous studies^6,17,30^. Additionally, Pana et al*.* have reported invasive mucormycosis in 9.5% of their study population with no underlying medical condition^34^. These findings are comparable to those of our study, and the difference in the results may be due to the difference in the patient population. In total, mucormycosis in immunocompetent individuals has a worldwide distribution regarding the analysis of literature reviews. Furtheremore, our results support the data previously reported that cutaneous mucormycosis and rhino-orbito-cerebral form are the most prevalent forms among patients with no underlying conditions^35^.

In the children population, our results are in agreement with a previous study by Pana et al. who have declared that hematologic malignancies are a significant risk factor for mucormycosis in children^34^.

It is noteworthy that almost a third of our children population (n:11, 28.9%) is immunocompetent hosts. These results support the data previously reported by Amanati et al.^36^ and Nidhi et al.^37^ that reported orbital and gastrointestinal mucormycosis in immunocompetent children, respectively.

The way of getting infection or transmission of infection in immunocompetent individuals is not clearly known. In this regards, a recent study have reported cases of mucormycosis in immunocompetent children with previous exposure to plants and fodder in infected children^36^. Evidence explains that mucormycosis mostly can be acquired by inhalation of sporangiospores, wherase ingestion or direct inoculation of spores can lead to gastrointestinal or cutaneous infections. Insect bites, stings, and even bird pecking have all been linked to cases of cutaneous mucormycosis in the past^38^. Therefore, determining the precise case histories in future investigations may reveal new aspects of epidemiology of mucormycosis.

Other clinical forms as gastrointestinal (n:1, 5.6%) was observed in immunocompetent patients in this study. Therefore, possible host and pathogen factors or mode of transmission the spores which can cause infection in individuals without predisposing conditions should be investigated in future studies.

The host immune system and portal of fungi entry may be responsible for the type of infection presentation. Similar to other studies, rhino-orbito-cerebral involvement was the most common form of infection. Also, in accordance with other previous studies, rhino-orbito-cerebral involvement is the most common clinical manifestation in hematologic malignancy and diabetic patients^17,19,23,26,39^. Regarding the distribution of spores in soil and decayed materials, inhaled sporangiospores can deposit in the nose or paranasalsinuses resulting in rhino-orbito-cerebral mucormycosis.

Cutaneous mucormycosis arise as a result of inoculation of spores in the skin layers or blood dissemination from infected organs. Although blood dissemination to skin tissue is rare, spore inoculation can result in cutaneous forms after trauma, burn, or iatrogenic injury. Although cutaneous manifestation has been reported as one of the common clinical forms of mucormycosis in previous studies,especially in immunocompetent individuals^6,17,19,31^, no case of cutaneous involvement was reported in our study. In this regards, it should be declared that separate hospitals provide pertinent services for trauma and burnning that were not included in this study.

In this study, clinical features were one of the most important pieces of evidence which contributed to the diagnosis of infection in all cases. According to our data, most patients with rhino-orbito-cerebralmucormycosis showed clinical signs such as facial pain, swelling, neurologic disorders, nasal ulcer, etc. Hence, vision and facial nerve disorders, the presence of a necrotic scar in maxillary, facial, or sino-orbital tissues in patients with uncontrolled diabetes, immunocompromised status, and, hematological malignancy could alert the physicians in order to early diagnose mucormycosis^40^. Furthermore, the presence of the nodule, mass, halo sign, ground-glass opacity, and the air crescent sign is radiologic evidence of respiratory/gastrointestinal tract fungal infections which should be remembered in the diagnosis of pulmonary/gastrointestinal mucormycosis^41^.

It should be noted that facial nerve palsy, an uncommon clinical manifestation in patients with rhino-orbito-cerebral mucormycosis regarding previous reports, has been announced in 2 patients in our study population^42^.

Histopathological examination of clinical specimens is the only laboratory method used in the diagnosis of all patients in this study that confirms the mucormycosis. It is worth noting that the histopathology method has still considerable value in the diagnosis of fungal infections. Observation of the non-septate or ribbon-like hyphae in tissue using histopathological examination classifies the infection as proven according to European Organisation for Research and Treatment of Cancer/ Mycoses Study Group (EORTC/MSG) criteria for mucormycosis infection^43^. At the same time, deformation of fungal hyphae due to various factors such as pressures exerted on the fungi by the tissue or folding during processing could result in artificial lines confused with septations and misidentification of fungal agents^41^. On the other hand,species identification and antifungal susceptibility profiles of fungal agents are valuable aspects in infectious diseases to identify the possible resistant species and geographical distribution of causative agents which is possible using culture of clinical samples.No information about the culture of patients' clinical samples was recorded in the medical record. According to a previous study, culture is performed to diagnose about 69% of cases, worldwide^6^. Since 2% of patients were diagnosed based on the culture method in Iran according to the previous investigation^17^, it is concluded that the culture method is not widely used to diagnose mucormycosis in Iran which requires more consideration.

Treatment guidelines documented by the European Society of Clinical Microbiology and Infectious Diseases (ESCMID) recommend surgical resection in addition to immediate antifungal therapy with liposomal or lipid-complex amphotericin B for adults and children with mucormycosis ^41^. Also, posaconazole is recommended for patients with drug intolerance and salvage therapy to prevent recurrence or combination therapy^41,44,45^.

Most of the patients in the present study received LAMB (86%) considering the first-choice antifungal for management of mucormycosis. Evidence by Jeong*et al*. also declared that initial monotherapy with liposomal amphotericin B is an effective approach to control mucormycosis and decrease mortality^46^. Given the low mortality rate of patients in this study, appropriate antifungal treatment accompanied by surgical intervention lead to improved outcomes in agreement with previous studies^30,47–49^. Indeed, drug penetration into the damaged tissue enhances by removing the necrotic tissue by surgical excision which causes the effective function of treatment. However, some previous studies have shown that surgery does not significantly affect decreased mortality in patients with mucormycosis^50^.

At the same time, the combination of LAMB plus azoles or caspofungin has been prescribed for a few patients. Antifungal therapy with LAMB plus caspofungin represents a promising successful treatment for rhino-orbito-cerebral mucormycosis in a study done by Reed et al.^51^. However, previous research shows that combination therapy did not result in less mortality in comparison with LAMB monotherapy^46^.

According to a study done by Jeong et al., there is no difference in survival between lipid-based formulation and deoxycholate formulation of amphotericin B^46^. However, it is not clear which form of amphotericin B has been used in this study; lipid formulation is more favorable due to less toxicity and side effects^52^.

In our study, the average dose of LAMB administered in children was 5–10 mg/kg/day. Due to the lack of defined optimal dosage of LAMB in children,the average dosage is often extrapolated from adult trials. In this regard, the recommended dosage by studies is 3–15 mg/kg/day^53^.

Posaconazole is another broad-spectrum triazole with significant antifungal activity against Mucorales fungi^54^. As a consequence of the unavailability of posaconazole in our country during recent years which leads to low levels of consumption in our study, it was not possible to compare the efficacy of posaconazole for the management of mucormycosis in adults and children. Moreover, isavuconazole is another therapeutic agent that is not available in our country's pharmaceutical market.

According to our results, the in-hospital mortality rate was 14.6%. However, the overall mortality rate of mucormycosis has been reported to range from 21.4 to 46.7% in previous studies^20,55,56^. Low in-hospital mortality rate has been reported in some earlier studies^14,57,58^.

Low mortality rate compared to other studies may be due to several reasons: (1) The reported mortality rate in our study is based on the in-hospital mortality, and excludes mortality data following discharge. As previously declared by Multani and their collegeues, 1-year mortality rate increased significantly compared to in-hospital mortality^58^.Therefore, if patients could have been followed up for a longer period of time, the mortality rate might have increased. (2) Lower mortality rates have been observed in cutaneous infections; therefore, the variable mortality rates reported in different studies may be related to the site of infection^57^. (3) Underlying conditions plays a crucial role in mortality rate of mucormycosis. As previously stated, immunocompetent status, or absence of trauma were related to the highest survival rates^41^. Therefore, a low mortality rate has been observed in this study might be a result of the small number of HSCT patients and CNS mucormycosis among our research population. (4) In addition, it is possible that some of the cases of mucormycosis that leads to death, might not have been properly diagnosed.

In our study, the children mortality rate was zero. In addition to those mentioned above, prematurity of the neonate, age less than 1 year, and the development of the disseminated form of mucormycosis have also been declared as risk factors for mortality in several studies conducted in the population ˂ 18 years^18,34,59^. In our investigation, no case of premature neonates or disseminated mucormycosis were found in the children's group. Besides, lack of reported in-hospital mortality among children may be linked to high proportion of immunocompetent conditions of studied population.

Various major limitations must be acknowledged in this study. In the current study, mortality information is confined to hospital lenght of stay. Moreover, diagnosis of mucormycosis was performed based on clinical features and histological examination. As a result, there was no specific culture methods for species identification and antifungal susceptibility testing of isolates. Hence, there is no available data about species distribution or resistance patterns of causative agents. Another limitation is the unavailability of such drug formulation in our country as isavuconazole,making it practically impossible to evaluate the efficacy ofthis treatment.

Although further explorations are needed to evaluate the efficacy of new therapeutic agents against fungal infections, it could be claimed that amphotericin B has been an effective antifungal for the management of mucormycosis, so far. With the hope of increasing awareness of fungal infections, the culture of clinical specimens, antifungal susceptibility testing, and molecular-based methods should give more attention as a particular subject in the diagnosis and management of fungal infections.

As far as we know, the current study is the first survey pointing to the epidemiological aspects, clinical presentation, therapeutic interventions, and outcomes of mucormycosis with a comparison between adults and children in Southwest Iran during the 8 years (2013–2021). A change in the epidemiology of mucormycosis has occurred in recent years due to increasing at-risk populations, especially in developing countries. In this study, diabetes mellitus and hematological malignancies were found as the most predisposing condition. Also, the rhino-orbito-cerebral clinical type was the most frequent form in patients. Early diagnosis and prompt clinical intervention may resulted in increased survival rate.

## Methods

### Patients and study setting

After being approved by the Ethics Committee of Shiraz University of Medical Sciences (IR. SUMS.MED.REC.1399.321), this multicenter retrospective study was performed from March 2013 through April 2021 in four main referral centers for infectious diseases in Shiraz, Iran (Namazi, Shahid Faghihi, Khalili and Amir hospitals) which affiliated to Shiraz University of Medical Sciences, Shiraz, Iran. These hospitals provide specialty and subspecialty services such as surgical, internal medicine, hematology/oncology, and ophthalmology/ENT for children and adults. Namazi and Shahid Faghihi are subspecialty hospitals as the largest referral medical centers in the south of Iran with 824 and 479 beds, respectively, and the average annual admission is more than 50,000/year. Also, Amir and Khalili hospitals as a specialized center for hematology/oncology and ophthalmology/ENT patients with 108 and 86 beds, respectively. In addition, all methods were performed in accordance with the relevant guidelines and regulations, and informed consent was obtained from all subjects and/or their legal guardians.

### Study protocol and definition

As defined by the European Organisation for Research and Treatment of Cancer/Mycoses Study Group (EORTC/MSG) criteria, all children and adults admitted and managed in studied centers with proven mucormycosis based on histopathology reports were included^43^. All registered data with diagnosis of mucormycosis, zygomycosis, or *Mucorales* infection were reviewed. Also, the data were reevaluated by infectious disease specialist in order to prevent data missing. The patients with a length of hospital stay fewer than 48 h because of not eligibility to follow up the clinical outcomes or patients with unavailable/incomplete data were excluded from the study. In data collection process, patients with admission in different centers were considered as one patient according to identical national code.

Then, all demographic information including sex, age, season/year of infection, underlying condition, and comorbid diseases as well as clinical data such as type of mucormycosis (site of infection), clinical presentation, radiological findings, laboratory-based diagnostic findings, surgical interventions, type, dose and duration of antifungal treatment, and clinical outcomes were extracted from the electronic database of hospitals by two physicians.

The annual incidence of mucormycosis was calculated annually by dividing the number of cases by the total number of hospitalizations observed in all centers in the same year.

### Statistical analysis

Collected data were analyzed by SPSS software (IBM Corp. Released 2013. IBM SPSS Statistics for Windows). Measurement data were expressed as mean ± standard deviation, as appropriate. For categorical variables in 2 × 2 tables, if there was a cell with expected frequencies of less than five, Fisher’s exact test was used. However, if there was not such a cell, the Chi-squared test with continuity correction was used. Comparisons were performed using Student’s t-test for continuous variables. It should be mentioned that a p-value of less than 0.05 was considered statistically significant.

## Data Availability

All data analysed during this study are included in this published article.
